# [^18^F]FDG PET/MRI in the follow-up of hepatocellular carcinoma after liver transplantation

**DOI:** 10.1097/MNM.0000000000001518

**Published:** 2022-01-11

**Authors:** Pietro Zucchetta, Carmelo Lacognata, Francesca Girardi, Alessandro Spimpolo, Filippo Crimì, Giulio Cabrelle, Chiara Zanon, Patrizia Boccagni, Laura Evangelista, Diego Cecchin, Umberto Cillo

**Affiliations:** a Department of Medicine – DIMED, Nuclear Medicine Unit, University of Padua; b Radiology Department, Azienda Ospedaliera di Padova, Padua; c Medicina Nucleare, Dipartimento di Diagnostica per Immagini, Azienda Sanitaria Universitaria Giuliano Isontina, Trieste; d Department of Medicine – DIMED, Radiology Unit; e Department of Surgery, Hepatobiliary Surgery and Liver Transplant Center, Oncology and Gastroenterology (DISCOG), University of Padua, Padua, Italy

**Keywords:** HCC, liver transplantation, PET/MRI

## Abstract

There is limited evidence regarding the application of [^18^F] fluorodeoxyglucose (FDG)-PET/MRI in patients with a suspected clinical recurrence, who underwent liver transplantation for hepatocellular carcinoma (HCC). Therefore, we compared the accuracy of PET/MR and standard-of-care (SOC) imaging in these patients.

**Methods:**

We retrospectively reviewed 26 patients, whose liver were transplanted for HCC and were suspected of disease relapse based on biochemical analysis or SOC follow-up imaging, and carried out PET/MRI with diffusion-weighted imaging sequences on them. All patients underwent SOC imaging within the 2 months prior to the PET/MRI examination and had follow-up data for at least 12 months after. Reference standards were histopathology, clinical and imaging follow-up data.

**Results:**

Sensitivity, specificity, positive predictive value, negative predictive value and accuracy for PET/MRI were 100, 94, 91, 100 and 96%, whereas for SOC imaging were 80, 69, 61, 85 and 73%. The accuracy of PET/MRI was higher with respect to SOC imaging, although not significantly.

**Conclusions:**

PET/MRI is useful for oncological surveillance of patients who have undergone liver transplantation for HCC, particularly in cases of allergy to contrast media, renal failure or persistently elevated alpha-fetoprotein levels, and with no identification of metastatic/relapsing foci at standard-of-care imaging.

## Introduction

Liver transplantation has been increasingly used in carefully selected patients affected by hepatocellular carcinoma (HCC) [1], because it eliminates the malignancy as well as the usually underlying cirrhosis, and ultimately restores normal liver function. However, despite state-of-the-art selection criteria for liver transplantation access, HCC recurrences after liver transplantation occur in 6–20% of patients, shortening their survival time to roughly 2 years [2–5]. The most common sites of recurrence include the liver alone (16% of cases), both the liver and distant organs (31%) and extra-hepatic regions alone (53%) [6].

There are currently no guidelines for post-liver transplantation surveillance imaging, due to a scarcity of literature on the subject [7]. Most centers perform surveillance every 3 months in the first year, every 6 months in the second and every 6–12 months in the 3rd to 5th years [6]. Noncontrast thoracic computed tomography (CT) and contrast-enhanced MR/CT of the abdomen are the standard-of-care (SOC) imaging modalities for surveillance, whereas liver ultrasound is a valuable alternative in patients allergic to contrast media or affected by renal failure [6,7]. MRI has been shown to be the most accurate diagnostic methodology for identifying HCC foci in both normal and cirrhotic livers; it has a detection rate of 68% with specific gadolinium-based contrast-enhanced sequences, increasing to as much as 90% when augmented with diffusion-weighted imaging (DWI) [8–11].

The role of [^18^F] fluorodeoxyglucose (FDG) PET in HCC diagnosis is less well established because HCC cells have a highly variable metabolism. FDG uptake is known to be dependent on the expression levels of GLUT1 and GLUT2 in the cell outer membrane. Furthermore, hexokinase enhances the accumulation of this radiotracer inside the tumor, phosphorylating it to [^18^F]FDG-6-phosphate [12–14]. Once phosphorylated, it cannot be further metabolized in the glycolysis pathway and is therefore ‘trapped’ in the cell. According to the most recent guidelines for the management of HCC issued by the European Association for the Study of the Liver, FDG uptake was observed in less than 40% of HCC patients before liver transplantation [15]. At the same time, low expression of GLUT1 and GLUT2, high expression of P-glycoprotein (a known drug efflux pump that may also act as an efflux pump for FDG), and high activity of FDG-6-phosphatase were demonstrated in moderately- and well-differentiated HCC [16]. However, many authors have reported the high sensitivity of FDG PET in detecting extra-hepatic HCC metastases [17–21], and its high diagnostic accuracy in assessing HCC viability after transcatheter arterial chemoembolization [22]. Moreover, FDG PET showed strong prognostic value in HCC patients, since the less well-differentiated and more aggressive HCCs usually have higher rates of glucose consumption [23,24].

Integrated PET/MRI imaging has recently been introduced in clinical practice in several tertiary, highly specialized centers, offering the unique opportunity of combining the advantages of MRI, which include increased soft-tissue contrast, lack of ionizing radiation exposure and availability of a wide range of sequences and contrast media, with the metabolic characterization of tissues provided by PET. The performances of these latest generation scanners are being explored in different clinical settings and may be particularly valuable for liver transplantation patient surveillance. Hence, the aim of this study was to compare the accuracy of FDG PET/MRI with SOC imaging in patients with suspected clinical recurrence who had undergone liver transplantation for HCC.

## Materials and methods

This single-center retrospective observational study was carried out in accordance with the Declaration of Helsinki guidelines and with approval from the Local Ethics Committee (protocol number: AOP1673 - 4831/AO/20). All patients participating in the study gave written informed consent to undergo FDG PET/MRI and to have their data accessed for scientific purposes.

### Patient selection

We retrospectively evaluated FDG PET/MRI studies of patients who had previously undergone liver transplantation for HCC and were subsequently referred to our department between September 2015 and March 2019 due to suspected disease relapse.

Inclusion criteria were as follows: availability of FDG PET/MRI data, SOC imaging studies performed within the previous 2 months and follow-up data covering a period of at least 12 months after the PET/MRI examination.

### Image acquisition

All FDG PET/MRI imaging was performed using a 3T Biograph mMR scanner (Siemens, Erlangen, Germany).

In accordance with European Association of Nuclear Medicine guidelines [25], patients were instructed to fast for at least 6 h before the intravenous injection of 3 MBq/kg of FDG and to rest for 60 min following injection to ensure proper tracer uptake.

After bladder voiding, integrated PET-MRI whole-body images were acquired with a dedicated radiofrequency body-coil, starting from the mid-thighs and continuing to the top of the head. The whole-body protocol included CAIPIRINHA-accelerated T1-weighted Dixon 3D-VIBE sequences for anatomical localization of the PET findings and attenuation correction of the PET data.

During the acquisition of the PET images, a whole-body dedicated MRI protocol was performed that included the following sequences: T2-weighted half-Fourier acquisition single-shot turbo spin-echo in the axial plane, T1-weighted spin-echo in the coronal plane, T1-weighted spoiled gradient recalled sequence with fat signal suppression (VIBE) in the axial plane, and spin-echo single-shot echo-planar diffusion weighted imaging (DWI) with *b* values of 0, 500 and 1000 s/mm^2^ in the axial plane.

PET data were reconstructed using 3D ordered subsets expectation maximization iterative algorithm with 3 iterations, 21 subsets and a 4-mm Full Width at Half Maximum Gaussian filter.

SOC imaging of patients with preserved renal function consisted in either contrast-enhanced thoracoabdominal CT or contrast-enhanced chest-CT plus contrast-enhanced upper abdomen MRI. Patients with chronic renal insufficiency instead underwent a chest-CT and abdominal ultrasound.

A 128-slice CT scanner (Somatom Definition, Siemens, Erlangen, Germany) was used for the CT imaging, and contrast-enhanced scans were performed after intravenous injection of 2 ml/kg of Iohexol 350 mg I/ml (Omnipaque, GE Healthcare, Milwaukee, USA) followed by a 50 ml saline flush.

Ultrasound examinations were performed with a Hitachi Ascendus Model EZU-MT28-SI (Hitachi, Tokyo, Japan).

A 1.5 Tesla MR scanner (Magnetom, Siemens, Erlangen, Germany) was used for the SOC MRI examinations with a protocol consisting of the following sequences: T2-weighted turbo spin-echo (TSE); T2-weighted TSE with fat saturation; in-phase and out-of-phase gradient echo; DWI with *b* values of 0, 500 and 1000 s/mm^2^; VIBE before and 25, 70 and 180 s after intravenous injection of a 0.2 ml/mg dose of 0.5 mmol/ml gadoteric acid (Dotarem, Guerbet, Roissy, France) followed by a 20 ml saline chaser, to obtain arterial, venous and equilibrium phase images, respectively.

### Image analysis

Images were analyzed and postprocessed on a dedicated workstation (SyngoVia, Siemens, Erlangen, Germany). A first-team, comprising a radiologist with 12 years’ experience, and a nuclear medicine physician with 10 years experience jointly evaluated all the FDG PET/MRI images; both were blind to patients’ clinical data and previous SOC imaging findings. The images were evaluated qualitatively and lesions showing either a qualitatively abnormal FDG uptake compared with the background or restricted water diffusion on DWI sequences were deemed ‘positive’.

A second team, comprising two radiologists, one with 10 years and one with 15 years experience, evaluated the SOC imaging data independently and blinded to the other team’s findings. When either local recurrences, lymph-nodal involvement, or distant metastases were detected, the patient was considered ‘positive’.

### Standard of reference

Histopathologic, clinical and imaging follow-up data were the gold-standard reference tests used to evaluate the diagnostic accuracy of PET/MRI and SOC imaging. A patient-based approach was taken, meaning that each individual was considered ‘positive’ when at least one lesion was identifiable on the images and ‘negative’ when no lesions were detectable. This was decided to avoid overrating the performance of one methodology if it revealed sites of metastasis/recurrence that the other did not.

### Statistical analysis

Sensitivity, specificity, accuracy, positive predictive value (PPV) and negative predictive value (NPV) were calculated for both FDG PET/MRI and SOC imaging using standard methods. SOC imaging and PET/MRI results were compared using the McNemar’s test, with the level of significance set at *P* <0.05. Statistical analyses were performed with the MedCalc software (MedCalc Software, Ostend, Belgium).

## Results

### Patients

A total of 26 patients were retrospectively enrolled in the study (24 males and 2 females; average age: 62 years; age range: 51–73 years). Seventeen patients had not been diagnosed with any recurrences/metastases since liver transplantation, whereas nine patients had already experienced disease recurrence after transplantation, either local recurrence (six patients), regional lymph node metastasis (*n* = 1) or distant metastases (*n* = 2). FDG PET/MRI examination was performed in 14 patients with suspicious findings on previous SOC imaging, and in the remaining 12 patients who had increasingly elevated alpha-fetoprotein (AFP) levels. SOC imaging consisted of contrast-enhanced thoracoabdominal CT of 15 patients, contrast-enhanced abdominal CT plus MR of 3, abdominal MR of 2 and abdominal ultrasound plus thoracic CT of 6 patients. Seven patients had stage IV or V chronic renal insufficiency at the time of SOC imaging, so no contrast medium was injected. Three patients presented deterioration in renal function in the period between SOC and FDG PET/MRI, so gadolinium-based contrast medium injection was no longer possible. Overall, at the time of FDG PET/MRI, eight patients had chronic renal insufficiency, three were allergic to the contrast medium and one had both conditions (Table 1).

**Table 1:** Study population characteristics. Chronic renal insufficiency is defined as having an eGFR of less than 30 ml/min/1.73 m2 (CKD 4 and 5) according to The European Society of Urogenital Radiology’s Guidelines on Contrast Media

Number of patients	26
Male	24
Female	2
Patient age (years)	
average	62
range	51-73
Post-liver transplantation treatments	
No treatment	17
Local treatment	6
Metastases surgery/RT	2
Lymph node RT	1
SOC imaging	
Abdomen CT and thorax CT	14
Abdomen MR	2
Abdomen CT and abdomen MR	3
Abdomen ultrasound and thorax CT	7
18F-FDG PET/MRI clinical question	
HCC relapse	7
HCC onset	14
LN relapse	2
HCC metastases	3
Functional renal status	
Chronic renal insufficiency	9
Normal	17
Contrast media allergy (Gd and iodine)	
Not allergic	22
Allergic	4

HCC, hepatocellular carcinoma.

### Standard-of-care and FDG PET/MRI

The reference standards were histopathological examination of the biopsy or surgical specimen in six patients, and follow-up imaging in the remaining 20.

The median time between PET/MR and SOC imaging was 29.5 days (range 1–60 days).

FDG PET/MRI had a sensitivity of 100% (10/10), specificity of 94% (15/16), PPV of 91% (10/11), NPV of 100% (15/15) and accuracy of 96% (25/26), whereas SOC imaging had a sensitivity of 80% (8/10), specificity of 69% (11/16), PPV of 61% (8/13), NPV of 85% (11/13) and accuracy of 73% (19/26). Considering only six patients who underwent ultrasound plus thoracic CT as SOC imaging, the accuracy of SOC imaging was 83% compared to a 100% accuracy of PET/MRI, whereas in the remaining 20 patients who underwent MRI or CT the accuracy of SOC imaging was 75% compared to a PET/MRI accuracy of 95% (Tables 2 and 3).

**Table 2:** Standard-of-care and FDG PET/MRI imaging performance

Performance (%)	SOC (95% CI)	18F-FDG PET/MRI (95% CI)	*P* value
Sensitivity	80 (44–97)	100 (69–100)	0.146
Specificity	69 (41–89)	94 (70–100)	0.073
Positive predictive value	61 (42–78)	91 (60–98)	0.099
Negative predictive value	85 (60–95)	100 (NA)	0.086
Accuracy	73 (52–88)	96 (80–100)	**0.023**

CI, confidence interval; SOC, standard-of-care.

**Table 3:** Standard-of-care and fluorodeoxyglucose PET/MRI performance by patient

Patient n.	CRI	CM Allergy	SOC	Provisional SOC diagnosis	FDG PET/MRI	FU
1	Yes	No	Ultrasound Abd + CT Tho	Local relapse + adrenal met.	Concordant	Local relapse + adrenal met.
2	Yes (after SOC)	No	CT Abd + CT Tho	Local relapse	Discordant – negative	Negative
3	No	No	CT Abd + CT Tho	LN relapse	Concordant	Negative
4	Yes	No	Ultrasound Abd + CT Tho	Negative	Concordant	Negative
5	No	No	CT Abd + CT Tho	Negative	Discordant – adrenal met.	Adrenal met.
6	No	No	CT Abd + MR Abd	New HCC + muscular met.	Concordant	New HCC + muscular met.
7	Yes	No	Ultrasound Abd + CT Tho	Local relapse	Concordant	Local relapse
8	No	No	CT Tho Abd	Negative	Concordant	Negative
9	Yes (after SOC)	No	CT Abd + MR Abd	Bone met. (T8 + S2)	Concordant + C2 met.	Bone met. (T8 + S2 + C2)
10	Yes	No	Ultrasound Abd + CT Tho	Negative	Discordant – lung relapse	Lung relapse
11	No	No	CT Tho Abd	Local relapse	Discordant – negative	Negative
12	Yes	No	Ultrasound Abd + CT Tho	Negative	Concordant	Negative
13	No	Yes	CT Tho Abd	Negative	Discordant – local relapse	Local relapse
14	No	Yes	CT Abd + MR Abd	Local relapse	Concordant	Local relapse
15	No	Yes	CT Tho Abd	Local relapse + bone mets.	Concordant	Local relapse + bone mets.
16	No	No	CT Tho Abd	Local relapse	Concordant	Local relapse
17	No	No	MR Abd	Local relapse	Discordant – negative	Negative
18	No	No	CT Tho Abd	Negative	Concordant	Negative
19	No	No	CT Tho Abd	Adrenal gland met.	Concordant	Adrenal gland met.
20	No	No	CT Tho Abd	Negative	Concordant	Negative
21	No	No	CT Tho Abd	Negative	Concordant	Negative
22	Yes	No	Ultrasound Abd + CT Tho	Negative	Concordant	Negative
23	Yes (after SOC)	Yes	MR Abd	Negative	Concordant	Negative
24	No	No	CT Tho Abd	Negative	Concordant	Negative
25	No		CT Tho Abd	LN relapse	Discordant – negative	Negative
26	No		CT Tho Abd	Negative	Concordant	Negative

CRI, chronic renal insufficiency; CM, contrast medium; FDG, fluorodeoxyglucose; HCC, hepatocellular carcinoma; SOC, standard-of-care.

Although the accuracy of PET/MRI was higher compared to that of SOC imaging, McNemar’s test did not show statistically significant differences between the two techniques (difference 8%; 95% confidence interval (CI), −10/+26; *P* = 0.6875).

**Fig. 1:**
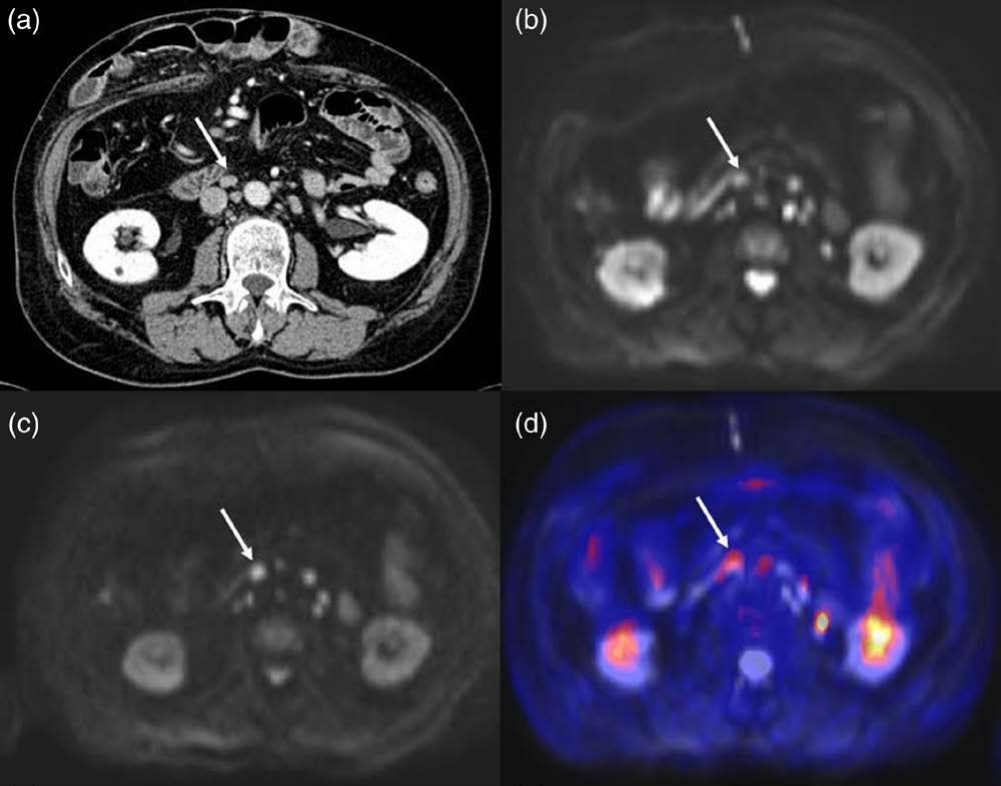
Contrast-enhanced CT scan showing enlarged inter-aorto-caval lymph node (arrow) with a short axis of 11 mm (a); PET/MRI showing the same lymph node in MRI-DWI b50 (b) and b1000 (c) sequences, with a focal uptake of fluorodeoxyglucose (FDG) (arrow) with a SUV_max_ of 5.4 (d). At histopathology after removing the largest lymph node, no malignant cells were detected. SUV, standardized uptake value.

Both PET/MRI and SOC imaging identified one patient (ID no. 3) as metastatic. This individual had several inter-aorto-caval lymph nodes with a short axis >10 mm and contrast enhancement on CT (Fig. 1a), which were also hypermetabolic (Standardized Uptake Value (SUV)_max_ 5.4) and DWI-restricted at FDG PET/MRI. After an excisional biopsy of one of the biggest nodes, only inflammatory alterations were found at histopathology; therefore, both FDG PET/MRI and SOC imaging wrongly classified the patient as metastatic.

**Fig. 2:**
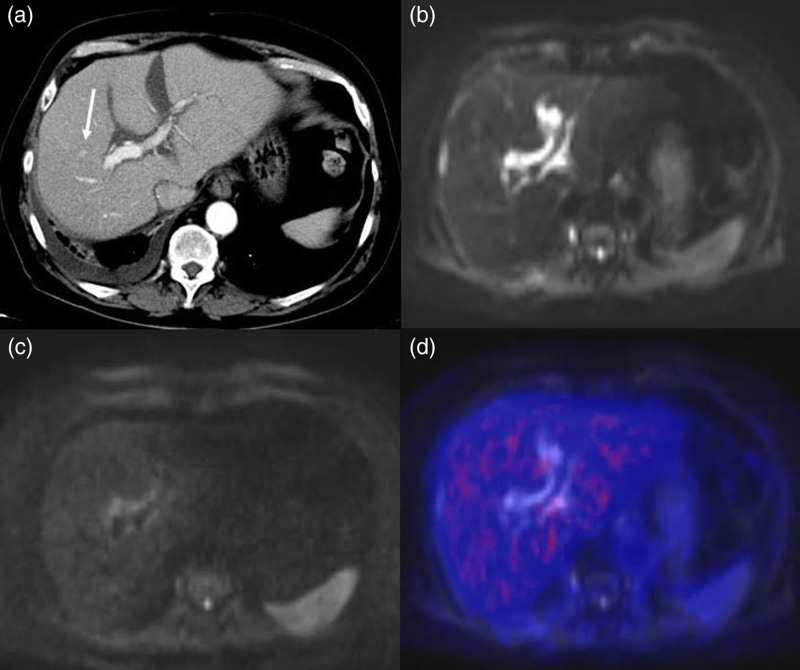
Contrast-enhanced CT scan showing a hypervascular nodule (arrow) in the S8 segment of the liver (a), suspect for hepatocellular carcinoma (HCC) recurrence; the lesion showed no restriction in the DWI sequences (b,c) nor hypermetabolism in the PET/MR fused images (d). At follow-up, it was confirmed to be a benign lesion.

**Fig. 3:**
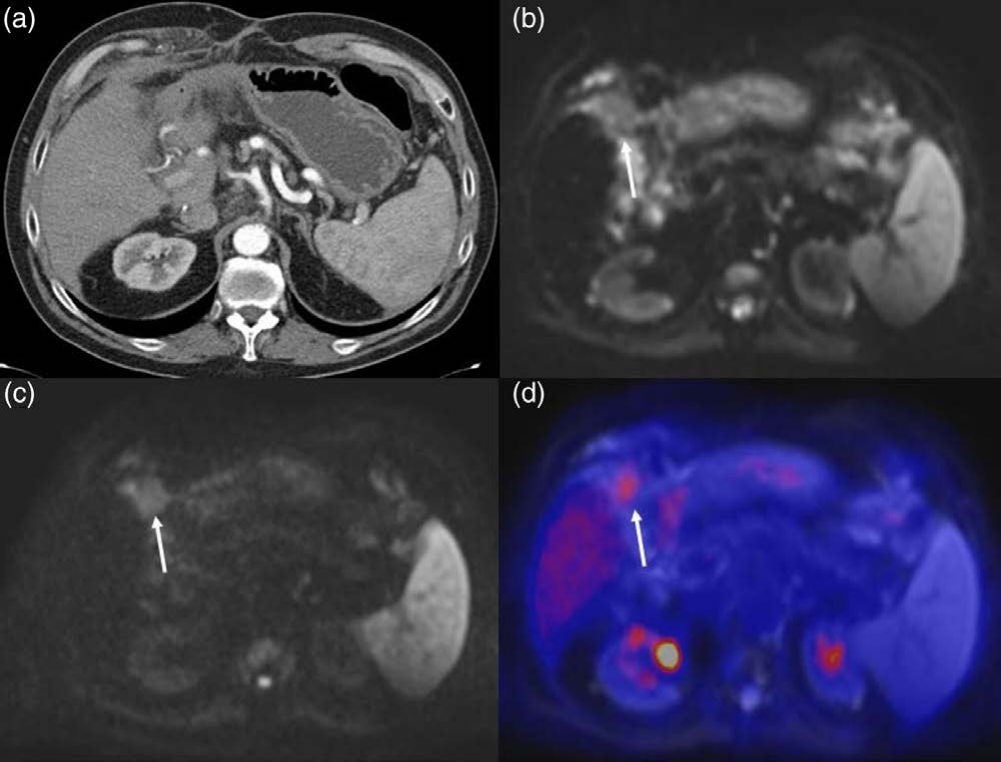
In a patient with persistently elevated alpha-fetoprotein levels, contrast-enhanced CT was not able to identify any recurrences (a), while PET/MRI detected an area of signal restriction (arrow) in the S4b segment of the liver (b,c), showing radiotracer uptake (arrow) at PET/MR fused images with a SUV_max_ of 4.9 (Fig. 3b–d). At biopsy, the lesion was confirmed to be an hepatocellular carcinoma (HCC) recurrence. SUV, standardized uptake value.

**Fig. 4:**
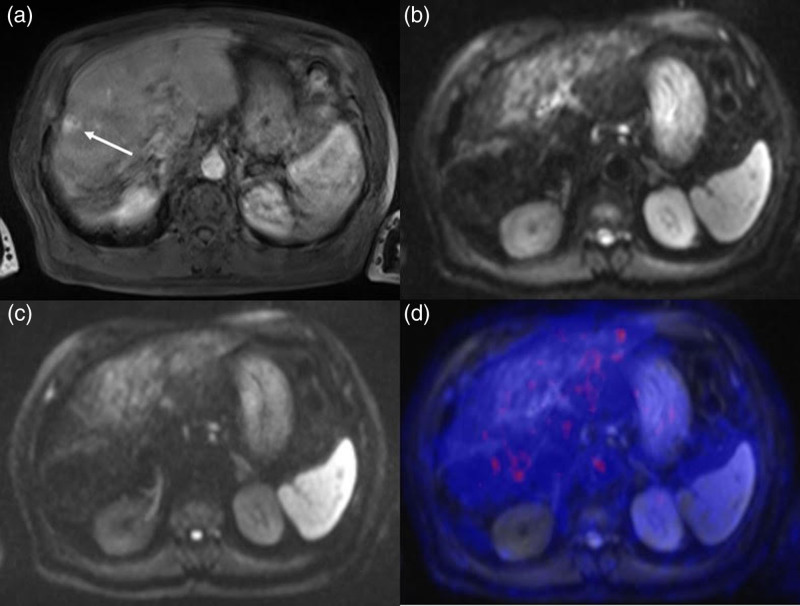
Contrast-enhanced MRI showing a hypervascular hepatic lesion in arterial phase (arrow), suspect for hepatocellular carcinoma (HCC) recurrence (Fig. 4a), but the lesion showed no diffusion restriction nor pathological hypermetabolism on fused PET/MRI images (b–d). Follow-up imaging confirmed that the lesion, which was classified as an arteriovenous fistula, was stable in size.

**Fig. 5:**
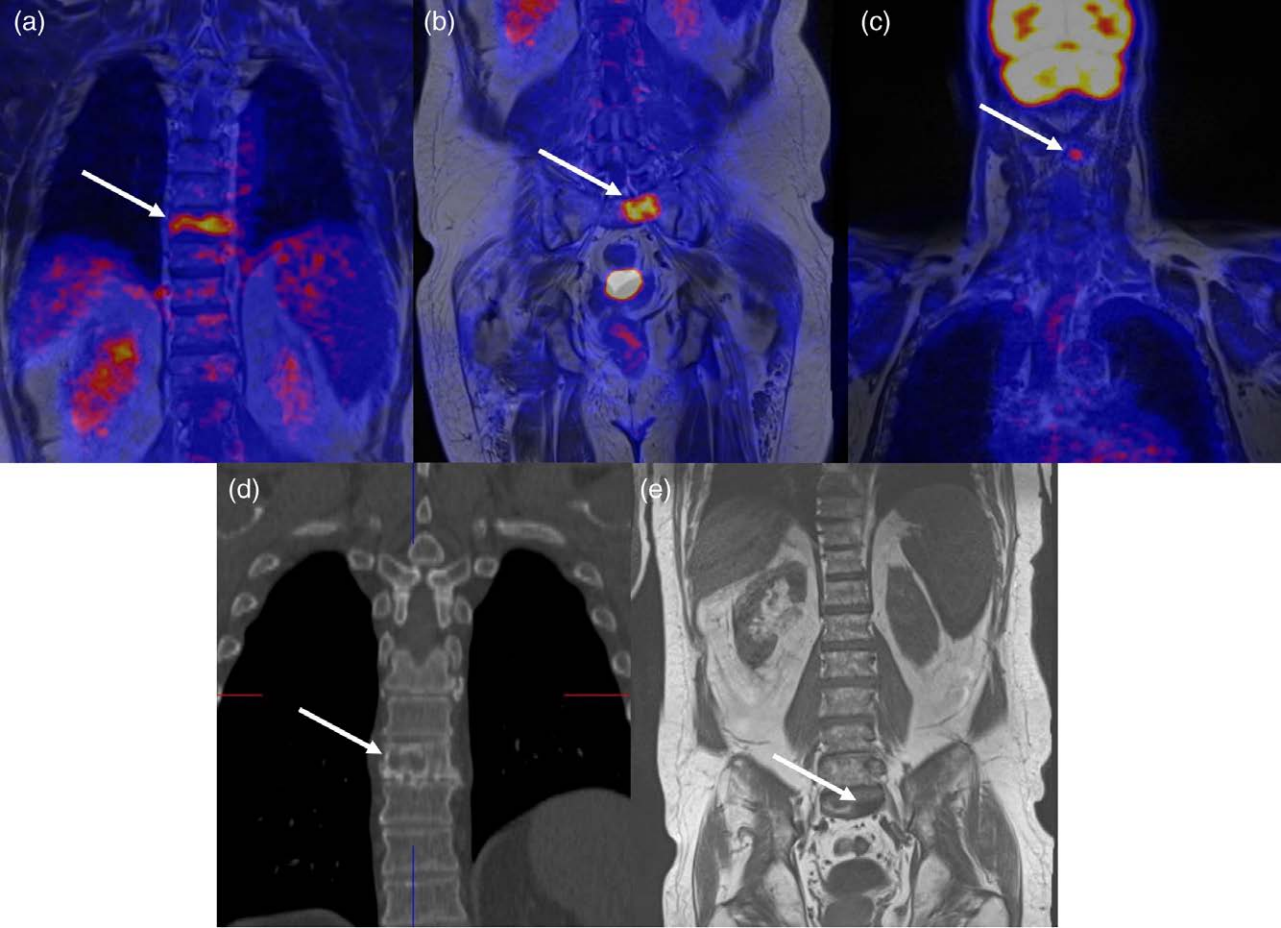
PET/MRI showing three hypermetabolic bone metastases (arrows) in the dorsal spine (a), sacrum (b), and cervical spine (c); (d) CT coronal reconstruction showing the osteolytic lesion (arrow) in the dorsal spine; (e) coronal T1 MR scan showing the bone metastasis (arrow) in the sacrum. The bone metastasis in the cervical spine was not identified by thoracic CT plus abdominal MRI, as it was out of the standard field of view.

Among the discordant cases, one patient (ID no. 2) showed a region of arterial-phase contrast enhancement followed by portal-phase washout in S8, which was interpreted as a hyper-vascularized nodule suspicious for HCC recurrence on the CT scan (Fig. 2a). FDG PET/MRI found neither 18F-FDG hypermetabolism nor any restriction of diffusivity in that area (Fig. 2b–d), so the patient was classified as ‘negative’, a diagnosis that was confirmed at follow-up imaging. In another patient (ID no. 13) with persistently elevated AFP levels and CT unable to identify any recurrences (Fig. 3a), FDG PET/MRI detected a hypermetabolic nodule in the liver (Fig. 3b–d) subsequently confirmed to be an HCC recurrence at histopathology. In another discordant case (ID no. 17), a hyper-vascularized hepatic lesion in the arterial phase was considered a recurrence on SOC contrast-enhanced abdomen MR (Fig. 4a), but it showed no diffusion restriction nor pathological hypermetabolism on FDG PET/MRI; subsequent imaging studies up to 1 year later detected no variation in size or signal intensity of the lesion, which was classified as an arteriovenous fistula. It is worth noting that in one of the concordant positive cases (ID no. 15), SOC imaging identified two bone metastases in T8 and S2 metamers, whereas FDG PET/MRI was able to detect an additional lesion site at the C2 level, a region that is not normally included in the SOC field of view (Fig. 5).

With regard to the subgroup of patients with chronic renal injury at the time of SOC (7/26 individuals), FDG PET/MRI and SOC were concordant and correct in 5/7 patients, and discordant in the remaining 2. In one of these two cases, PET/MRI identified a lung metastasis that CT had misclassified as a scar, and in both these cases, PET/MRI excluded a provisional diagnosis of liver relapse made on ultrasound images.

## Discussion

In our study, FDG PET/MRI was more accurate than SOC imaging in detecting recurrences in patients who had undergone liver transplantation for HCC.

As far as we know, FDG PET/MRI has not been found to have a better diagnostic performance than MRI at HCC staging [26], probably because of the low sensitivity of FDG, especially in cases of well-differentiated tumors [15]. Other radiotracers, such as 11C-Acetate, 11C-Choline and its 18F-labeled derivatives, have proved to be more accurate than FDG, particularly in the staging of primary hepatocellular malignancies [27].

Nevertheless, when HCC metastases or local recurrences after treatment are suspected, FDG PET/CT has displayed good accuracy, probably because of undifferentiated components of the tumoral lesion. FDG PET has therefore already been recommended in these clinical scenarios [17–21,28]. In particular, Vermersch *et al*. [28] confirmed the superiority of PET/MRI respect to the combined analysis of CT and liver MRI for HCC M-staging; moreover, PET/MRI led to changes in the therapeutic management in almost 10% of patients detecting additional metastases or by reducing the uncertainty regarding metastatic involvement [28].

The combination of FDG PET imaging and MRI with DWI, which has already been shown to improve HCC detection compared with traditional MRI sequences [8–11], was found to have a high diagnostic value in our study. With the PET/MRI methodology, it is, in fact, possible to gather functional information regarding DWI and FDG, linking cellularity to metabolism, in the same examination, allowing precise identification of the metastatic foci.

The use of FDG PET/CT along with conventional radiologic imaging (i.e. contrast-enhanced CT) has already been recommended in cases where there is high suspicion of recurrence in patients who have previously undergone liver transplantation for HCC [29]. A meta-analysis of studies with sample sizes ranging from 11 to 31 patients showed FDG PET/CT to have a sensitivity of 81.7% and a specificity of 88.9% in detecting HCC recurrences [21]. In our study, we found PET/MR to have 100% sensitivity and 94% specificity in detecting HCC recurrences after liver transplantation, showing that this technique is better than or at least comparable to PET/CT in this clinical setting. Since PET/MR has been shown to subject patients to less radiation exposure than PET/CT (mean dose reduction: 50%; range: 18.9–64.3%) [30], these results show PET/MR to be a useful tool in the follow-up of liver-transplanted patients.

Moreover, if we compare PET/MR with conventional radiological imaging, the wider field of view that this technique affords, encompassing the head and neck regions without exposing the patient to additional radiation, allowed us to detect in a single study a further metastasis located in the cervical vertebral body that had been missed by the previous traditional contrast-enhanced thoraco-abdominal CT.

The use of FDG PET and DWI together obviated the need for gadolinium injection in patients with impaired renal function or allergies. Indeed, a group of patients in our study were not able to undergo contrast-enhanced imaging, such as CT or MRI, due to renal failure. The European Society of Urogenital Radiology’s current guidelines advise that to avoid the risk of acute kidney injury or systemic nephrogenic fibrosis, caused by iodine-based and gadolinium-based contrast media, respectively, these agents should not be used in patients with an estimated glomerular filtration rate (eGFR) below 30 ml/min/1.73 m^2^ and to use them carefully in patients with an eGFR in the range 30–45 ml/min/1.73 m^2^ [31]. Moreover, in fragile liver transplantation patients, who have already suffered the nephrotoxic effects of immunosuppressive treatments, the repeated use of intravenous contrast media can further worsen renal function, and it has been shown that patients who had previously suffered an acute kidney injury resulting from contrast medium administration have a higher likelihood of developing renal failure later on [32].

FDG, on the other hand, is known to be well-tolerated in patients at risk of renal failure and does not place unnecessary stress on potentially impaired kidneys. In the specific setting of liver transplantation, the role of FDG PET/MRI is therefore even more important, as there is no risk of worsening renal function, and accuracy is higher than either ultrasound, unenhanced CT or MRI alone. Moreover, FDG PET/MRI allowed us to correctly classify two patients in our study population with renal insufficiency, who had been misdiagnosed by previous SOC imaging. In one case, a liver relapse had been suspected, but regular FDG uptake and apparent diffusion coefficient (ADC) values were concordant in excluding that diagnosis. In another patient, a fibrotic lesion in the lung parenchyma had been deemed metastatic by SOC imaging, but FDG uptake and ADC values typical of fibrotic changes were absent at FDG PET/MRI.

The intrinsic limitations of MRI in depicting lung parenchyma reduces the accuracy of PET/MRI compared with CT in detecting small pulmonary lesions [33–35], even though new sequences have been recently developed to address this issue [36,37]. This is certainly worth taking into account when performing PET/MRI, because at present it can only be partially fixed by using nonroutine sequences.

Our study presents some limitations first, it is retrospective and involves a relatively small sample of patients. Second, SOC imaging comprised different techniques (although it resembled the ‘real word’ clinical setting), ranging from ultrasound to MRI, which made the comparison with PET/MRI more challenging. Moreover, the long interval between SOC and PET/MRI (the longest 60 days) could have introduced a bias in the comparison, because lesions that were not detectable at the time of SOC imaging could have grown, becoming more easily identifiable when PET/MRI was performed.

Nevertheless, the results suggest that FDG PET/MRI should be considered as a tool for oncological surveillance of patients who have undergone liver-transplantation for HCC, particularly in cases of allergy to contrast media, renal failure or persistently elevated AFP levels, and with no identification of metastatic/relapsing foci at standard-of-care imaging.

The next step in using PET/MR in the follow-up of patients affected by HCC who have undergone liver transplantation could be to use a radiotracer with greater HCC sensitivity, such as - but not only - radio-labeled 11C-choline.

## Acknowledgements

**Conflicts of interest**

There are no conflicts of interest.
